# Comparison of model-averaging and single-distribution approaches to estimating species sensitivity distributions and hazardous concentrations for 5% of species

**DOI:** 10.1093/etojnl/vgae060

**Published:** 2025-01-06

**Authors:** Yuichi Iwasaki, Miina Yanagihara

**Affiliations:** Research Institute of Science for Safety and Sustainability, National Institute of Advanced Industrial Science and Technology (AIST), Tsukuba, Ibaraki, Japan; KWR Water Research Institute, Nieuwegein, the Netherlands; Center for Marine Environmental Studies, Ehime University, Matsuyama, Ehime, Japan

**Keywords:** ecological risk assessment, species sensitivity distributions, aquatic toxicology, environmental quality benchmarks, Akaike information criterion (AIC)

## Abstract

Estimation of species sensitivity distributions (SSDs) and hazardous concentrations for 5% of species (HC5s) by fitting a statistical distribution to toxicity data for multiple species is essential in ecological risk assessment of chemicals. Given the challenge of selecting the appropriate statistical distribution in SSD estimation, a model-averaging approach that involves fitting multiple statistical distributions and using weighted estimates to derive HC5s is appealing. However, the effectiveness of this approach compared with SSDs based on a single statistical distribution (i.e., single-distribution approach) has not been thoroughly examined. We aimed to compare the model-averaging approach with the single-distribution approach based on log-normal, log-logistic, Burr type III, Weibull, and gamma distributions to estimate HC5s. For this comparison, we selected 35 chemicals with available toxicity data for more than 50 species, enabling the direct calculation of reference HC5 values from the 5^th^ percentiles of the toxicity distributions. For each chemical, we examined the deviations between the reference HC5 value and HC5 estimates derived from SSDs based on toxicity data for 5–15 species subsampled from the complete dataset using model-averaging and single-distribution approaches. This subsampling simulated the typical limitations of available toxicity data. The deviations observed with the model-averaging approach were comparable with those from the single-distribution approach based on the log-normal, log-logistic, and Burr type III distributions. Although use of specific distributions often resulted in overly conservative HC5 or HC1 estimates, our results suggest that the precision of HC5/HC1 estimates would not substantially differ between the model-averaging approach and the single-distribution approach based on log-normal and log-logistic distributions. We further discuss the circumstances under which model-averaging and single-distribution approaches are better suited for estimating HC5s.

## Introduction

The use of species sensitivity distributions (SSDs) for assessment of the ecological risk of chemicals is essential in deriving defensible “safe” concentrations and quantifying community-level effects resulting from exposure by extrapolating impacts on multiple species from toxicity data for individual species (Belanger et al., 2017; Forbes & Calow, 2002; Fox et al., 2021; Posthuma et al., 2002; Posthuma et al., 2019; Schipper et al., 2014). There are generally two approaches for estimating SSDs: fitting of parametric statistical distributions and nonparametric estimation. Nonparametric estimation, such as calculating percentiles directly from raw data, requires toxicity data for a large number of species, which are unavailable for most chemicals. The former method, fitting parametric statistical distributions to toxicity data obtained from multiple species, has thus been commonly used. A hazardous concentration for 5% of species (HC5) is then estimated and used to derive “safe” concentrations such as environmental quality benchmarks (e.g., standards, criteria, or guidelines).

Many statistical distributions, such as log-normal, log-logistic, Weibull, and Burr type III distributions, have been used to estimate SSDs (Fox et al., 2021; Newman et al., 2000; Yanagihara et al., 2024), and no single universally applicable distribution has been determined. Although Yanagihara et al. (2024) demonstrated that the choice of the statistical model often does not significantly affect the HC5 estimates, selecting the appropriate distribution remains a challenge in SSD estimation. To address this challenge, a model-averaging approach has been used to estimate SSDs and thereby HC5s (Fox et al., 2021; Schwarz & Tillmanns, 2019) and has been used in several studies (e.g., Binet et al., 2024; Hamoutene et al., 2023; Wang et al., 2023). In model averaging, multiple statistical distributions are fitted to data, and the measure of “goodness of fit” (e.g., Akaike information criterion [AIC]; Akaike, 1973) is used to weight the estimates of, for example, the HC5. The application of model averaging to SSD estimation (hereafter referred to simply as the model-averaging approach) is promising because it does not require selecting a single distribution and incorporates the uncertainty in model selection (Schwarz & Tillmanns, 2019).

Despite its appealing features, model averaging does not necessarily guarantee a reduction in prediction error (see Dormann et al. 2018 for a more comprehensive discussion). Schwarz and Tillmanns (2019) have visually demonstrated that HC5 values estimated using the model-averaging approach are insensitive to the addition of toxicity data. Such insensitivity is not surprising given the original features of the method, but that analysis was based on only three chemicals. There have hence been few attempts to examine how effective this model-averaging approach is in predicting HC5 values.

The goal of this study was to compare the model-averaging approach with SSD estimation based on single statistical distributions (hereafter referred to as the single-distribution approach) to estimate HC5 values when toxicity data are available for a limited number of species. For this comparison, we first compiled complete datasets for selected chemicals with acute toxicity data available for a large number of species (*n* > 50) from an existing database. To simulate the data availability, we generated the subsampled datasets by selecting toxicity data for 5–15 species from the complete datasets. For each chemical, the reference HC5 value, directly calculated from the complete dataset, was compared with HC5 estimates derived from model-averaging and single-distribution approaches based on the subsampled datasets. Because the model-averaging approach takes into account the uncertainty associated with model selection, we hypothesized that model-averaging would reduce the large deviations from the HC5 values compared with the single-distribution approach.

## Materials and methods

### Data

All the ecotoxicity data used were collected from the EnviroTox database version 2.0.0, which has been curated from existing databases (https://envirotoxdatabase.org; Connors et al., 2019). Chronic toxicity data were not used in this study because of their limited availability; only one insecticide (chlorpyrifos) met the selection criteria outlined below. We selected 35 chemicals with acute toxicity data from the database based on the following criteria: (1) the effect measures were either the 50% effect concentration (EC50) or the median lethal concentration (LC50); (2) we excluded effect concentrations that exceeded five times the water solubility of the chemical tested (Connors et al., 2019); and (3) effect concentrations were available for more than 50 biological species from at least three of four taxonomic groups (i.e., algae, invertebrates, amphibians, and fish) for a given chemical. We required inclusion of at least three taxonomic groups based on the fact that regulatory frameworks require toxicity data from multiple taxonomic groups for estimating SSDs (Belanger et al., 2017). In addition, given that the optimal sample size for reliable nonparametric estimation has been determined to be 15–55 (Newman et al., 2000), we used a relatively conservative threshold of 50 species for selecting the chemicals to be examined. For the nonparametric estimate (i.e., an estimate made without assuming a specific statistical distribution), we obtained an HC5 value for each chemical by calculating the fifth percentile from the complete dataset. In this study, we refer to these HC5 values as reference HC5 values. In cases where multiple effect concentrations were available for an individual species and a chemical, we used the geometric mean in the SSD estimation.

Chemicals with specific modes of action can result in bi- or multimodal SSDs that necessitate the estimation of separate SSDs for groups with different sensitivities (Oginah et al., 2023). Although estimating separate SSDs was beyond the scope of this study, we used the sample size, skewness, and excess kurtosis to calculate the bimodality coefficient for the complete toxicity dataset (Fox et al., 2021). We took into consideration bimodality if the bimodality coefficient exceeded 0.555 for each chemical (Fox et al., 2021; Freeman & Dale, 2013; Pfister et al., 2013), and we subsequently examined how the bimodality affected our results. In addition, according to the classification in Kramer et al. (2024) and our additional investigation (See online supplementary material Table S1), 31 of the 35 chemicals were used as biocides or pesticides (hereafter simply referred to as biocides) such as fungicides, insecticides, and herbicides. To gain insights into the influence of the biocide use group, we categorized any chemical used as a biocide as a biocide even if it had other uses. The remaining four chemicals examined—sodium dodecyl sulfate, sodium nitrite, aniline, and acetone—were classified as industrial chemicals (See online supplementary material Table S1).

Furthermore, we analyzed freshwater and saltwater toxicity data together because no systematic differences have been observed between freshwater and saltwater SSDs estimated based on acute toxicity data for 104 chemicals in the EnviroTox database (Yanagihara et al., 2022). We excluded metals including copper, cadmium, and zinc from our analysis because of the challenges associated with considering the influence of water quality characteristics such as pH and hardness on the toxicity of these elements (Adams et al., 2020; Meyer et al., 1999), although the inclusion of these metals in our analysis did not materially affect our findings.

### Subsampled datasets for SSD estimation

To compare the performance of the single-distribution approach and the model-averaging approach, we generated 1,000 simulated datasets by randomly subsampling 15 species-specific geometric means without replacement from the complete dataset of each chemical. The rationale for this subsampling was to simulate the limited availability of toxicity data commonly encountered in practical ecological risk assessments. We ensured that the 15 subsampled species included species from at least three taxonomic groups according to the criterion (3) applied to the complete dataset. The subsampled datasets were used for subsequent SSD estimation based on the model-averaging and single-distribution approaches. We also performed complementary analyses by subsampling five or 10 species-specific geometric means and obtained results similar to those we obtained with 15 species (See below for more details).

It should be emphasized that the aim of this study was to compare the relative performance of the model-averaging and single-distribution approaches in HC5 estimation using reasonably realistic datasets rather than datasets that strictly follow regulatory jurisdiction requirements. When deriving environmental quality benchmarks or predicted no effect concentrations, more stringent requirements are generally adopted (e.g., the need for toxicity data from specific taxonomic groups and the separate use of freshwater and saltwater toxicity data), although such requirements vary across regulatory jurisdictions (Belanger et al., 2017; European Food Safety Authority Panel on Plant Protection Products and their Residues [EFSA PPR Panel], 2013). Among these requirements, for example, the taxonomic composition of species included in SSDs has been shown to have a significant influence on the HC5 estimation (Maltby et al., 2005; Oginah et al., 2023). Although the subsampled toxicity datasets generated based on our relaxed criteria can still include datasets that comply with the more stringent regulatory requirements, the observed variations in HC5 estimation do not necessarily reflect outcomes under such requirements.

### Species sensitivity distribution derivation

The derivation of SSDs and the estimation of HC5 were conducted using the “ssdtools” package (Thorley & Schwarz, 2018) in R version 4.3.1 (R Core Team, 2023). We fitted a total of five statistical distributions to the subsampled toxicity data for the 35 chemicals using maximum likelihood estimation. Based on a previous study (Yanagihara et al., 2024), we selected four frequently used statistical distributions (log-normal, log-logistic, Burr type III, and Weibull distributions) as well as the gamma distribution, which we also included in this study. The HC5 values for the single-distribution approach were then estimated by fitting the five statistical distributions to these data using the “ssd_fit_dists” and “ssd_hc” functions. Using these functions, we also obtained the HC5 estimate based on the model-averaging approach for each chemical by calculating a weighted average of the estimated HC5 values. The model weight was calculated from the difference (ΔAICc) between the corrected AIC for small sample sizes (AICc; Burnham and Anderson 2002; Burnham et al. 2011) and the best model (i.e., the model with the lowest AICc value; see *Equation 1*):


(1) $$w_{i}=\frac{\exp(-\frac{\Delta A\text{IC}c_{i}}{2})}{\sum_{k=1}^{K}\exp(-\frac{\Delta A\text{IC}c_{k}}{2})}$$

where *w_i_* represents the weight for model *i*, and *K* is the number of models (in this study, *K *=* *5). The R code and related files can be accessed at a GitHub repository at https://github.com/yuichiwsk/ssd_modelavg_examined.

Fitting the Burr type III distribution to the toxicity data often failed because of convergence issues. The median frequency (and range) of fitting failures for the Burr type III distribution was 439 (154–858) out of 1,000 subsampling iterations among the 35 chemicals. Fitting the Burr type III distribution using the maximum likelihood method is known to be susceptible to numerical instability and failure to converge (Fox et al., 2021). In our study, we simply excluded such cases from further analysis.

For each chemical, we then calculated the deviations (i.e., differences) between the reference HC5 value and HC5 estimates derived from the subsampled datasets using the model-averaging approach and the single-distribution approach (log-normal, log-logistic, Burr type III, Weibull, or gamma distributions). Specifically, for each approach, we compared the 2.5, 50, and 97.5 percentile deviations, which we calculated from deviations observed in 1,000 subsampled datasets for individual chemicals. In this analysis, the deviations were computed as the differences of the log_10_-transformed HC5 values. All deviations mentioned herein (*vide infra*) were calculated in this manner unless otherwise specified. For each chemical, the HC5 estimates from the model-averaging and single-distribution approaches varied among the 1,000 subsampled datasets, and the single reference HC5 value calculated from the corresponding complete dataset was considered to be the most likely HC5 estimate for the comparison.

## Results and discussion

### Comparison of deviations of HC5 estimates

**Figure 1.**
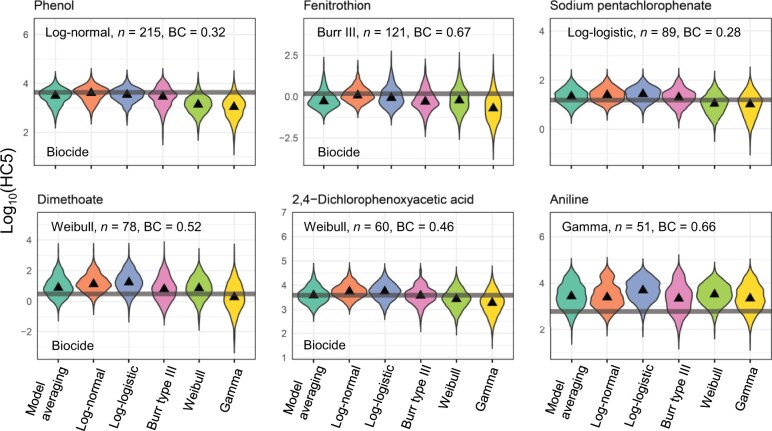
Hazardous concentrations of 5% of species (HC5) estimated using model-averaging and single-distribution (i.e., log-normal, log-logistic, Burr type III, Weibull, and gamma distributions) approaches for six chemicals. Subsampled datasets consisting of 15 species-specific toxicity data points were used to estimate each species sensitivity distribution. These six chemicals were selected as examples from 35 chemicals examined in the study (See online supplementary material Figure S1). Horizontal lines represent reference HC5 values directly calculated from the complete datasets. Black triangles indicate median values based on HC5 estimates from 1,000 subsampling iterations. Violin plots (kernel density estimates) illustrate the actual distributions. The distribution name in each panel is the best distribution with the lowest value of the corrected Akaike information criterion for the complete dataset. The designations “n” and “BC” indicate the number of species and the bimodality coefficient for the complete dataset, respectively.

Despite the collection of chemicals being biased towards those used as biocides, the deviations of the HC5 estimates for the single-distribution and model-averaging approaches differed very little between the biocides and four industrial chemicals (Figure 1; see online supplementary material Figure S1 for the complete set of chemicals). For example, both the overestimates and underestimates of HC5 values were independent of these categories. We therefore discuss the deviations of HC5 estimates without distinguishing between biocides and industrial chemicals.

**Figure 2.**
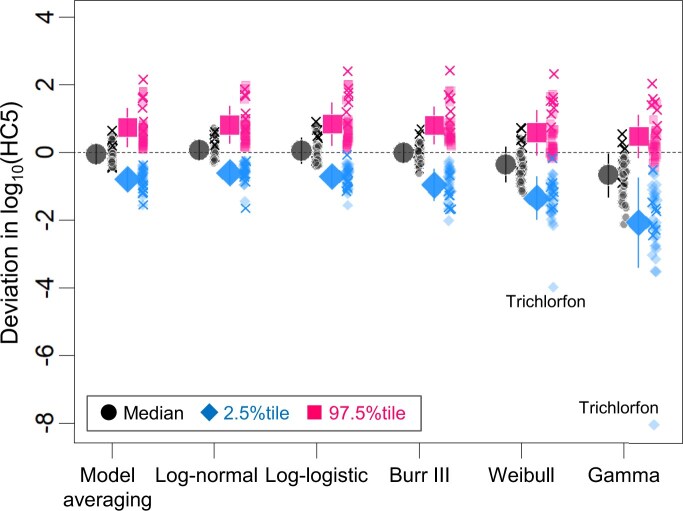
Deviations between reference hazardous concentrations of 5% of species (HC5) and those estimated using model-averaging and single-distribution approaches based on log-normal, log-logistic, Burr type III, Weibull, and gamma distributions. Subsampled datasets consisting of 15 species-specific toxicity data points were used to estimate each species sensitivity distribution. For each approach, the averages of the 2.5, 50, and 97.5 percentile values of deviations across 35 chemicals are shown with a large diamond, circle, and square, respectively, whereas individual values for these chemicals are represented by the corresponding small symbols. Crosses represent chemicals with bimodality coefficients greater than 0.555. The 2.5 and 97.5 percentile values are included to illustrate how each approach underestimates or overestimates the HC5 values. Error bars indicate ±1 SD.

The medians of the deviations between the HC5 values derived from the model-averaging approach and the reference HC5 values were close to zero (average of medians across 35 chemicals: −0.06; ranging from −0.5 to 0.7; Figure 2). Averages of their 2.5 and 97.5 percentile deviations were −0.8 (range: −1.6 to −0.4) and 0.7 (range: 0.1 to 2.2), respectively. These values and ranges were comparable to those observed with the single-distribution approach based on the log-normal, log-logistic, and Burr type III distributions (Figure 2). For example, for the log-normal SSDs, averages of the 2.5, 50, and 97.5 percentile deviations were −0.6 (range: −1.6 to −0.2), 0.08 (range: −0.3 to 0.7), and 0.8 (range: 0.1 to 2.0), respectively. Similar results were obtained when fewer (i.e., 5 or 10) species-specific data points were analyzed (See online supplementary material Figures S2 and S3). These results, based on the log-normal, log-logistic, and Burr type III distributions, did not support our initial hypothesis that the model-averaging approach would reduce the large deviations of HC5 estimates compared with the single-distribution approach (but see below for a more detailed discussion of SSDs based on the Weibull and gamma distributions).

This finding is not surprising given that the individual distributions generally predicted the HC5 values equally well, as evidenced by the deviations of HC5 estimates observed for the single-distribution approach based on the log-normal, log-logistic, and Burr type III distributions (Figure 2). Similarly, by fitting four statistical distributions (log-normal, log-logistic, Burr type III, and Weibull distributions) to acute and chronic toxicity data for 191 and 31 chemicals, respectively, collected from the EnviroTox database, Yanagihara et al. (2024) have demonstrated that the relative prediction accuracy (i.e., AICc values) and HC5 estimates from the log-normal SSDs are comparable with those from other SSDs in most cases. Yanagihara et al. (2024) have also found that the Weibull distribution tends to produce lower HC5 estimates than other distributions, and this trend was likewise observed in our study (Figure 2). We also observed that the gamma distribution tended to produce more conservative HC5 estimates (Figure 2), probably because its low shape parameters (< 1 in 32%–100% of cases across all chemicals, with a median of 99%) lead to a steep initial rise in these distributions. However, it is important to note that these lower HC5 estimates occasionally matched the reference HC5 values. For instance, the median HC5 estimate of the gamma distribution for dimethoate was the closest to the reference HC5 value (Figure 1). An interesting implication of the Weibull distribution’s being selected as the best for dimethoate was that the best distributions, selected based on the AICc from the complete datasets, did not always yield the closest HC5 estimate (See also online supplementary material Figure S1).

Seven chemicals had bimodality coefficients greater than 0.555 based on the complete datasets (Figure 1 and online supplementary material Figure S1); five of those chemicals (e.g., fenitrothion) were categorized as biocides; the remaining two were aniline and acetone. Whereas the 50 and 2.5 percentile deviations were not significantly affected by bimodality, chemicals that exhibited bimodality had larger 97.5 percentile deviations in all the model-averaging and single-distribution approaches (Figure 2; Welch’s *t*-tests with Holm’s adjustment, *p *<* *0.05). A notable example was aniline, where both the model-averaging and single-distribution approaches tended to overestimate HC5, as reflected in the median HC5 estimates that were 4–8 times the reference HC5 value (Figure 1). Daphniids such as *Daphnia magna*, *Daphnia pulex*, and *Ceriodaphnia dubia* are known to be highly sensitive to aniline (Ramos et al., 2002), and five toxicity values obtained for daphniids were characteristically lower than those for other species (See online supplementary material Figure S4). The overestimates of HC5 values observed for aniline would likely have occurred if these relatively low toxicity values were not included in the subsampled datasets. Estimating separate SSDs such as taxonomic-group-specific SSDs (Oginah et al., 2023), could be useful for addressing this overestimation issue, but acquiring sufficient toxicity data for individual taxonomic groups remains a critical challenge (Belanger et al., 2017; Johnson et al., 2020).

The results of this study did not reveal any remarkable advantages of the model-averaging approach compared with the single-distribution approach based on, for example, log-normal and log-logistic distributions in terms of deviations of HC5 estimates. Binet et al. (2024) have shown that model-averaging approaches reduce the occurrences of extremely low 99% protective concentrations (i.e., hazardous concentrations for 1% of species, HC1s) compared with the single-distribution approach based on Burr type III and inverse Pareto distributions, although this is not the case with the log-normal distribution. In the complementary analysis, where HC1 was estimated instead of HC5, we observed that the use of the Burr type III distribution yielded more conservative HC1 estimates than the model-averaging approach. The difference was pronounced in the case of 2.5 percentile deviations (See online supplementary material Figure S5). However, we did not observe such conservative estimates in SSDs based on log-normal and log-logistic distributions. These results did not contradict the findings of Binet et al. (2024), which were based on chronic toxicity data. Although acute toxicity data are used for SSD estimation for pesticides (EFSA PPR Panel, 2013), chronic toxicity data are preferred for estimating environmental quality benchmarks or predicted no effect concentrations; therefore, a thorough examination of our findings would be desirable by analyzing chronic toxicity data. Despite this caveat, given that the single-distribution approach based on the Weibull and gamma distributions could also lead to overly conservative HC5 estimates (Figure 2), our results suggest that use of a limited set of specific distributions, such as Burr type III, Weibull, and gamma, should be avoided both in the model-averaging and single-distribution approaches for estimating HC5 or HC1.

### Model averaging vs. single distribution in HC5 estimation

Our results suggest that the deviations of HC5 or HC1 estimates do not substantially differ between the model-averaging approach and the single-distribution approach based on log-normal and log-logistic distributions when only limited toxicity data (i.e., 5–15 species) are available. Based on this finding, the use of the model-averaging approach may be recommended over the single-distribution approach based on a log-normal (or log-logistic) distribution under the following circumstances. First, the model-averaging approach is useful for obtaining a semi-automated, reasonable estimation of HC5 based on a weighted compilation of various distribution models. Even if a distribution model does not fit the data well, it has little influence on the overall HC5 estimation. This characteristic is appealing to regulatory authorities because an HC5 can then be estimated without detailed considerations of the fitting of specific statistical distributions, especially if the use of software programs that implement the model-averaging approach, such as the U.S. Environmental Protection Agency’s SSD Toolbox (Etterson, 2020) and R’s ‘ssdtools’ package (Thorley & Schwarz, 2018), is permitted. Indeed, the R “ssdtools” package and its web application (“shinyssdtools”) are officially used in Canada and are planned for official use in Australia and New Zealand (Fox et al., 2023). Second, although Schwarz and Tillmanns (2019) tested only three chemicals, they have demonstrated that the model-averaging approach to HC5 estimation can be insensitive to the addition of toxicity data that might introduce unexpected variations in species sensitivity. Third, the model-averaging approach can be especially useful when the toxicity data are not well described by a log-normal (or log-logistic) distribution, but other distributions included in model averaging provide a substantially better fit. However, such cases are likely uncommon (Yanagihara et al., 2024). Even if the best model is not the log-normal distribution based on, for example, the AICc, the resulting differences in HC5 estimates may not be substantial (Yanagihara et al., 2024). Overall, given the fact that a single best distribution model cannot be determined a priori, the model-averaging approach will continue to play a beneficial role in SSD estimation.

In contrast, the single-distribution approach based on the log-normal (or log-logistic) distribution is reasonably pragmatic in cases where the use of model averaging or specific software tools is restricted, for example, by regulatory authorities. An appealing feature of using the log-normal or log-logistic SSD is that the estimation can easily be performed even by hand (Aldenberg et al., 2002). Furthermore, the accuracy of HC5 estimation can be theoretically and easily quantified, particularly for the log-normal distribution (i.e., the normal distribution with log-transformed effect concentrations), because its mathematical properties have been thoroughly investigated (Aldenberg & Jaworska, 2000; Aldenberg et al., 2002; Kamo et al., 2022; Sorgog & Kamo, 2019).

Although not examined in this study, the determination of the proportions of taxonomic groups included in an SSD (Duboudin et al., 2004; Hayashi & Kashiwagi, 2010; Maltby et al., 2005), and the handling of multiple toxicity data for a species (Duboudin et al., 2004) can influence SSD and HC5 estimation regardless of which of the two approaches is selected. The decision between the model-averaging approach and the single-distribution approach based on the log-normal (or log-logistic) distribution would be relatively insignificant compared with the above issues and would largely be influenced by restrictions on the use of software programs for model averaging, as our results suggest that the two approaches are generally comparable in HC5 estimation.

## Supplementary Material

vgae060_Supplementary_Data

## Data Availability

All data used in the present study are available from the EnviroTox database (https://envirotoxdatabase.org/). The corresponding R code and related files can be accessed at a GitHub repository (https://github.com/yuichiwsk/ssd_modelavg_examined).
