# Supplementary material for: Comparison of model-averaging and single-distribution approaches to estimating species sensitivity distributions and hazardous concentrations for 5% of species
Source: Environ Toxicol Chem. 2025 Jan 6;44(3):834–40. doi: 10.1093/etojnl/vgae060 (PMC11864202; doi:10.1093/etojnl/vgae060)
Supplement: vgae060_Supplementary_Data [file vgae060_supplementary_data.zip › vgae060_Supplementary_Data/Supporting_Information_Figures_rev1_v3.docx]

Supporting Information for

**Comparison of model-averaging and single-distribution approaches to estimate species sensitivity distributions and hazardous concentrations for 5% of species**

Yuichi Iwasaki^a^* and Miina Yanagihara^b,c^

^a^ Research Institute of Science for Safety and Sustainability, National Institute of Advanced Industrial Science and Technology (AIST), Tsukuba, Ibaraki, Japan

^b^ KWR Water Research Institute, Nieuwegein, the Netherlands

^c^ Center for Marine Environmental Studies, Ehime University, Matsuyama, Ehime, Japan

NOTE:

All data used in the present study are available from the EnviroTox database (<https://envirotoxdatabase.org/>). The corresponding R code and related files can be accessed at a GitHub repository (<https://github.com/yuichiwsk/ssd_modelavg_examined>).

**Figure S1:** Hazardous concentrations of 5% of species (HC5) estimated using model-averaging and single-distribution approaches based on log-normal, log-logistic, Burr type III, Weibull, and gamma distributions. Subsampled datasets consisting of 15 species-specific toxicity data points were used to estimate each species sensitivity distribution. Horizontal lines represent reference HC5 values directly calculated from the complete datasets. Black triangles indicate median values based on HC5 estimates from 1000 subsampling iterations. Violin plots (kernel density estimates) illustrate the actual distributions. The distribution name in each panel is the best distribution with the lowest value of the corrected Akaike information criterion for the complete dataset. The designations “n” and “BC” indicate the number of species and bimodality coefficient for the complete dataset, respectively.

**Figure S1** (continued)

**Figure S1** (continued)

**Figure S2:** Deviations between reference hazardous concentrations of 5% of species (HC5) and those estimated using model-averaging and single-distribution approaches based on log-normal, log-logistic, Burr type III, Weibull, and gamma distributions. Subsampled datasets consisting of five species-specific toxicity data points were used to estimate each species sensitivity distribution. For each approach, the averages of the 2.5, 50 (median), and 97.5 percentile values of deviations across 35 chemicals are shown with a large diamond, circle, and square, respectively, while individual values for these chemicals are represented by the corresponding small symbols. Crosses represent chemicals with bimodality coefficients greater than 0.555. The 2.5 and 97.5 percentile values are included to illustrate how each approach underestimates or overestimates the HC5 values. Error bars indicate ±1 standard deviation.

**Figure S3:** Deviations between reference hazardous concentrations of 5% of species (HC5) and those estimated using model-averaging and single-distribution approaches based on log-normal, log-logistic, Burr type III, Weibull, and gamma distributions. Subsampled datasets consisting of 10 species-specific toxicity data points were used to estimate each species sensitivity distribution. For each approach, the averages of 2.5, 50 (median), and 97.5 percentile values of deviations across 35 chemicals are shown with a large diamond, circle, and square, respectively, while individual values for these chemicals are represented by the corresponding small symbols. Crosses represent chemicals with bimodality coefficients greater than 0.555. The 2.5 and 97.5 percentile values are included to illustrate how each approach underestimates or overestimates the HC5 values. Error bars indicate ±1 standard deviation.


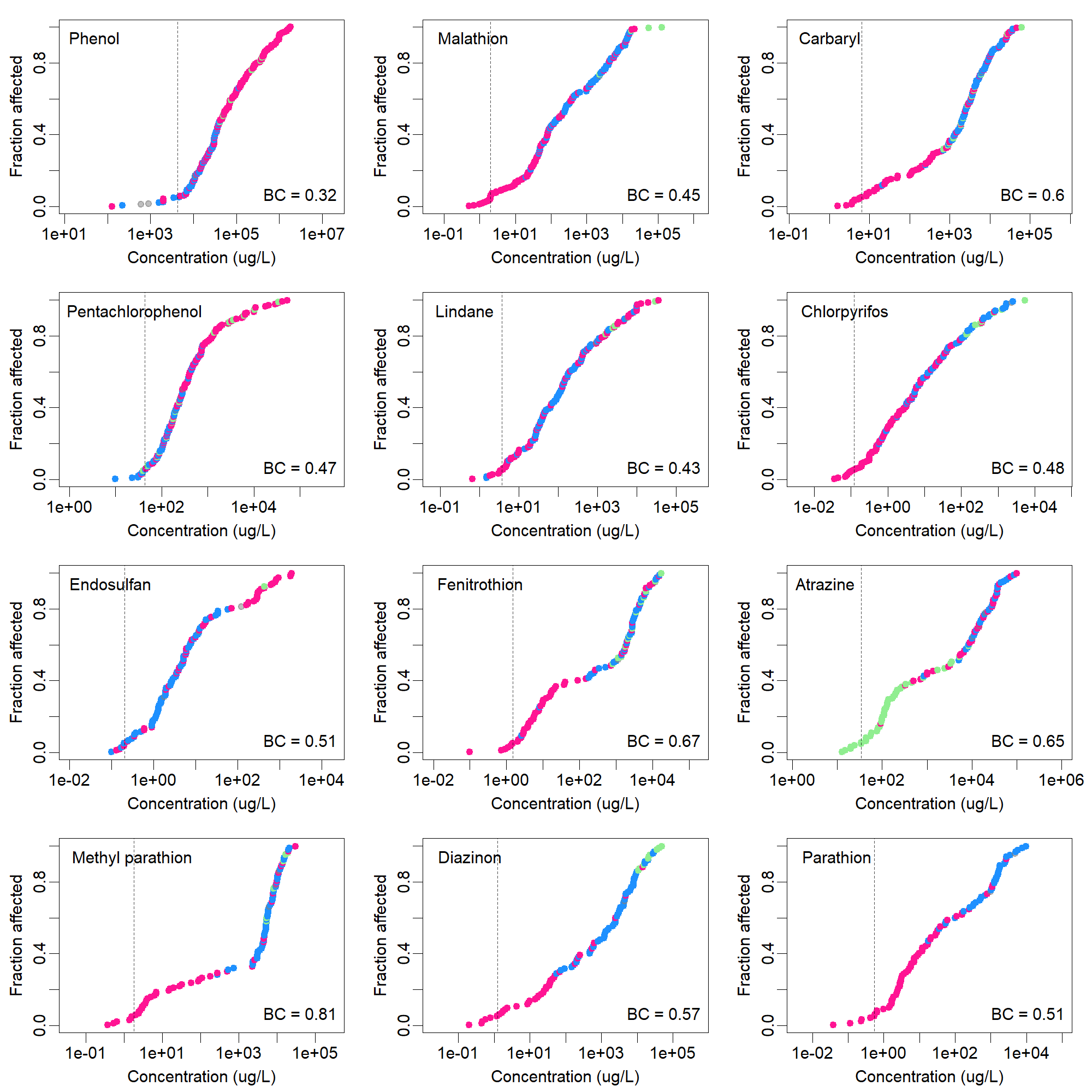


**Figure S4:** Species sensitivity distributions for 35 chemicals. Geen, pink, gray, and blue dots represent effect concentrations for algae, invertebrates, amphibians, and fish, respectively. Vertical dotted lines indicate the hazardous concentration of 5% of species (HC5) derived from the complete datasets (i.e., reference HC5 value). “BC” indicates a bimodality coefficient.

**Figure S4** (continued)

**Figure S4** (continued)

**Figure S5:** Deviations from reference hazardous concentrations of 1% of species (HC1) estimated using model-averaging and single-distribution approaches based on log-normal, log-logistic, Burr type III, Weibull, and gamma distributions. Subsampled datasets consisting of 15 species-specific toxicity data points were used to estimate each species sensitivity distribution. For each approach, the averages of the 2.5, 50 (median), and 97.5 percentile values of deviations across 35 chemicals are shown with a large diamond, circle, and square, respectively. Individual values for these chemicals are represented by the corresponding small symbols. Crosses represent chemicals with bimodality coefficients greater than 0.555. The 2.5 and 97.5 percentile values are included to illustrate how each approach underestimates or overestimates the HC1 values. Error bars indicate ±1 standard deviation.
